# Super resolution using sparse sampling at portable ultra-low field MR

**DOI:** 10.3389/fneur.2024.1330203

**Published:** 2024-05-24

**Authors:** Corinne Donnay, Serhat V. Okar, Charidimos Tsagkas, María I. Gaitán, Megan Poorman, Daniel S. Reich, Govind Nair

**Affiliations:** ^1^Translational Neuroradiology Section, National Institutes of Health, National Institute of Neurological Disorders and Stroke, Bethesda, MD, United States; ^2^Wellcome Centre for Integrative Neuroimaging, FMRIB, Nuffield Department of Clinical Neurosciences, University of Oxford, Oxford, United Kingdom; ^3^Hyperfine, Inc., Guilford, CT, United States; ^4^Quantitative MRI Core, National Institutes of Health, National Institute of Neurological Disorders and Stroke, Bethesda, MD, United States

**Keywords:** super-resolution, ultra-low field, multiple sclerosis, Fourier-transform, reconstruction

## Abstract

Ultra-low field (ULF) magnetic resonance imaging (MRI) holds the potential to make MRI more accessible, given its cost-effectiveness, reduced power requirements, and portability. However, signal-to-noise ratio (SNR) drops with field strength, necessitating imaging with lower resolution and longer scan times. This study introduces a novel Fourier-based Super Resolution (FouSR) approach, designed to enhance the resolution of ULF MRI images with minimal increase in total scan time. FouSR combines spatial frequencies from two orthogonal ULF images of anisotropic resolution to create an isotropic T2-weighted fluid-attenuated inversion recovery (FLAIR) image. We hypothesized that FouSR could effectively recover information from under-sampled slice directions, thereby improving the delineation of multiple sclerosis (MS) lesions and other significant anatomical features. Importantly, the FouSR algorithm can be implemented on the scanner with changes to the *k*-space trajectory. Paired ULF (Hyperfine SWOOP, 0.064 tesla) and high field (Siemens, Skyra, 3 Tesla) FLAIR scans were collected on the same day from a phantom and a cohort of 10 participants with MS or suspected MS (6 female; mean ± SD age: 44.1 ± 4.1). ULF scans were acquired along both coronal and axial planes, featuring an in-plane resolution of 1.7 mm × 1.7 mm with a slice thickness of 5 mm. FouSR was evaluated against registered ULF coronal and axial scans, their average (ULF average) and a gold standard SR (ANTs SR). FouSR exhibited higher SNR (47.96 ± 12.6) compared to ULF coronal (36.7 ± 12.2) and higher lesion conspicuity (0.12 ± 0.06) compared to ULF axial (0.13 ± 0.07) but did not exhibit any significant differences contrast-to-noise-ratio (CNR) compared to other methods in patient scans. However, FouSR demonstrated superior image sharpness (0.025 ± 0.0040) compared to all other techniques (ULF coronal 0.021 ± 0.0037, *q* = 5.9, *p*-adj. = 0.011; ULF axial 0.018 ± 0.0026, *q* = 11.1, *p*-adj. = 0.0001; ULF average 0.019 ± 0.0034, *q* = 24.2, *p*-adj. < 0.0001) and higher lesion sharpness (−0.97 ± 0.31) when compared to the ULF average (−1.02 ± 0.37, *t*(543) = −10.174, *p* = <0.0001). Average blinded qualitative assessment by three experienced MS neurologists showed no significant difference in WML and sulci or gyri visualization between FouSR and other methods. FouSR can, in principle, be implemented on the scanner to produce clinically useful FLAIR images at higher resolution on the fly, providing a valuable tool for visualizing lesions and other anatomical structures in MS.

## 1 Introduction

Magnetic resonance imaging (MRI) holds critical clinical importance in neuroradiology due to its exceptional ability to visualize soft tissue contrast; however, substantial financial barriers associated with purchasing, installing, and maintaining routinely used MRI scanners restrict easy access to MRI, especially in developing and underdeveloped countries (1). Recent advancements in ultra-low field (ULF) brain MRI (main magnetic fields less than 0.1 tesla) offer a compelling alternative to their high field (HF), high-cost counterparts (2). These advantages come at the cost of reduced image quality, including reduced contrast-and signal-to-noise ratios (CNR, SNR) associated with the lower main magnetic field.

Reduced SNR at low fields can be at least partially compensated by signal averaging over multiple acquisitions or, more often, by lowering the overall resolution in the scan. However, increasing the scan time through more signal averaging may lead to image degradation through patient motion, and lowering resolution can lead to missing small but potentially clinically important features in neuroimaging, such as the white matter lesions (WML) common in small vessel disease and multiple sclerosis (MS) (3). Acquiring multiple scans and combining them through super-resolution (SR) could potentially address the limitations of ULF MRI systems, as it can improve image quality beyond the native resolution, allowing for more detailed and sharper visualization of anatomical structures (3, 4).

One SR approach that has shown promise in improving the quality of ULF MRI images is interpolation-based SR methods, which estimate voxel values at subvoxel positions by averaging or interpolating neighboring voxel values, effectively increasing the resolution (4). This has been applied to ULF MRI by reslicing and averaging orthogonal acquisitions of simultaneously acquired T2 weighted and T2 maps (5) and orthogonal FLAIR acquisitions (3). Importantly, acquiring multiple planes to reconstruct a higher-resolution image dramatically increases scan time, which can compromise the clinical appeal of such methods. Alternatively, frequency domain SR approaches can enhance image resolution by transforming low-resolution images into the discrete Fourier transform (DFT) domain (6). This allows for precise estimation of motion parameters, including planar rotation and horizontal and vertical shift, through phase correlation analysis of spatially shifted images in the Fourier domain (7). Transforming MRI data into the frequency space is valuable as it separates low-frequency components, carrying contrast information, from high-frequency components containing high-resolution details. This approach proves especially beneficial for enhancing the resolution of aliased images, as low-frequency components are free from aliasing effects, enabling improved image registration before averaging (7).

In this work, we introduce an SR algorithm that combines spatial frequencies (Fourier-based SR or FouSR) obtained from ULF T2-weighted fluid-attenuated inversion recovery (FLAIR) brain images acquired along two orthogonal planes to produce an isotropic image of higher resolution, opening the possibility of performing SR on the fly during patient scanning. This method is similar in approach to the keyhole imaging technique implemented for dynamic scanning, where only the low-frequency components of successive images are collected to improve the time-resolution of bolus passage (8). Here, we use the high-frequency component of a second scan to improve spatial resolution and visualization of small features in a structural scan. We collected 0.064 T (64 mT) FLAIR images along orthogonal planes and paired 3 T FLAIRs on a phantom and 10 participants with MS. We hypothesized that the FouSR algorithm would effectively add information from the under-sampled slice directions with additional data within the ULF images, leading to enhanced delineation of MS lesions and other significant anatomical features. The results were quantitatively compared for SNR and CNR and visually rated.

## 2 Algorithm theory

The fundamental principle of Fourier imaging in MRI is that the spatial information of an object is encoded in the frequency domain during data acquisition and then reconstructed back into the spatial domain to form the final image. The relationship between 2-dimentional *k*-space and image space in MRI can be given as:


$$\rho \left(x,y\right)=\int \int S\left(k_{x},k_{y}\right)\exp\left(ik_{x}x+ik_{y}y\right)dk_{x}dk_{y}\text{,}$$

where $\rho \left(x,y\right)$ is the image in spatial domain, and S is the detected signal in the frequency domain (*k*-space). The signal is a complex valued-function that characterizes the amplitude and phase of the signal at each point in *k*-space and the expression $\exp\left(ik_{x}x+ik_{y}y\right)$ represents the spatial information encoded by the magnetic field gradients during the data acquisition process. The inverse 2D Fourier transform, denoted by ∫∫ integrates over all spatial frequencies, to recover the image.

**Figure 1:**
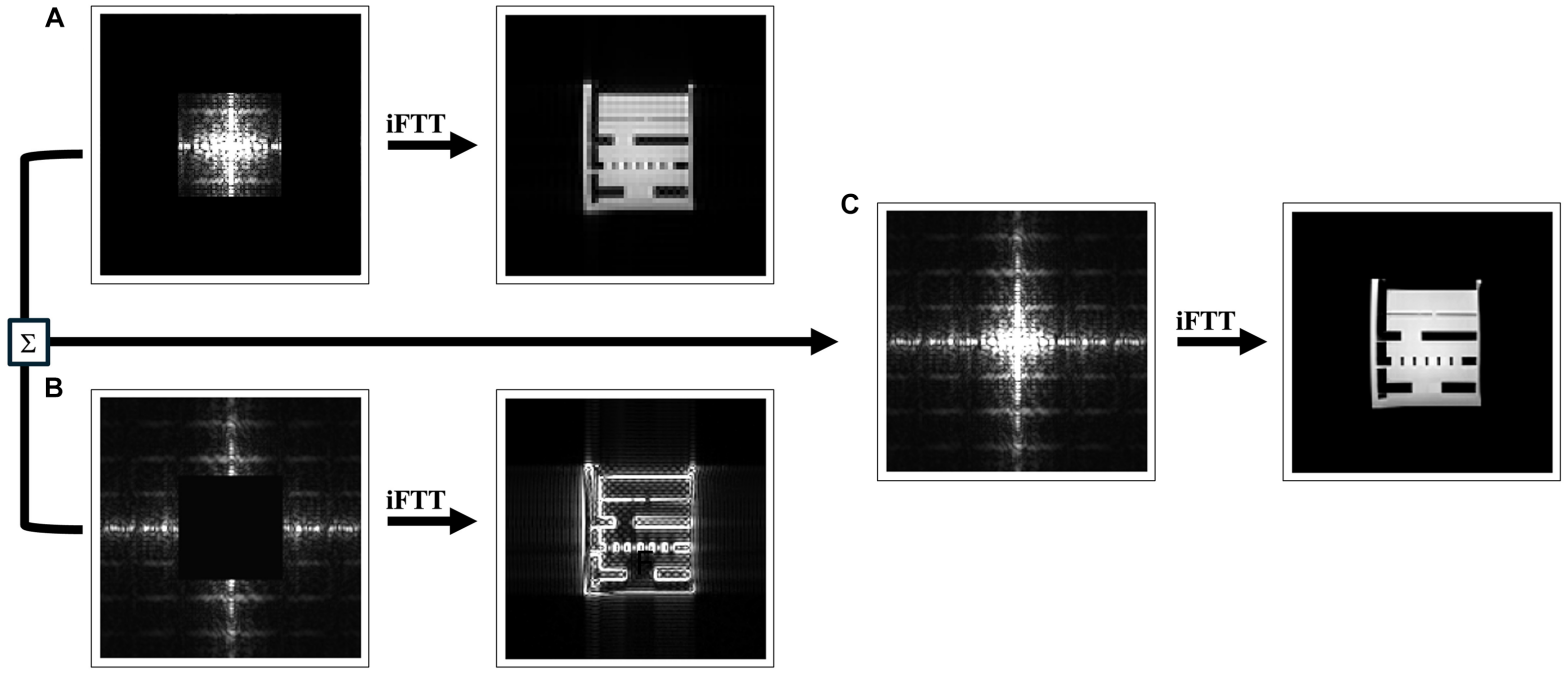
Examples of how sampling frequency influences the quality of MRI images. A coronal slice of a standard hyperfine phantom, scanned at 3T and downsampled to 1.6x1.6x1.6mm voxel sizes, was Fast Fourier transformed (FFT) to obtain frequency space data. The inverse FFT (IFFT) of **(A)** only the central portion of the frequency space produces a blurred image that is rich in contrast as seen in the phantom; **(B)** the periphery of the frequency space produces images with clear edges of the phantom but lacking in contrast between fluid and background; and **(C)** the full frequency space produces the highest quality images.

As shown in Figure 1, when only the center of frequency space, or low frequencies are retained, the resulting image preserves contrast but at the expense of spatial resolution, leading to a blurry appearance. When high frequencies are sampled, the resulting image preserves sharp edges but may sacrifice contrast resolution. In the case of an anisotropic 3D acquisition, with lower resolution through-plane than in-plane, one frequency-space extent along the slice direction would be limited. Additional high-frequency component data could be acquired by switching the scanning to an orthogonal plane. As more data is acquired in the periphery of the *k*-space, higher frequencies components in the image are added increasing the overall quality of the image (Figure 1).

## 3 Methods and materials

### 3.1 Data collection

**Figure 2:**
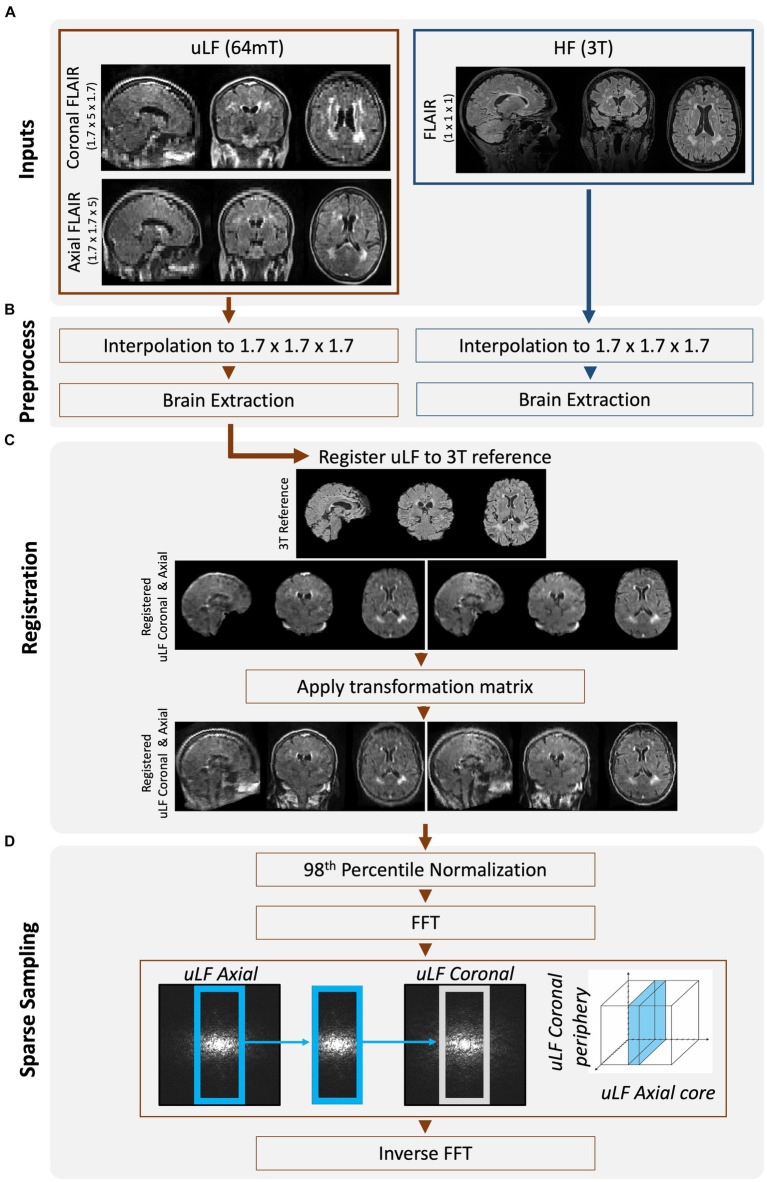
Overview of workflow to create FouSR images. **(A)** Same-day FLAIR scans were acquired at ULF (64 mT) and HF (3 T). ULF FLAIR images were acquired in 2 planes, coronal and axial, but are shown here in triplanar reformation. **(B)** All FLAIR images were interpolated to 1.7 mm isotropic. ULF images were brain extracted using SynthStrip (9). HF images were cropped to remove neck and BRAIN extracted using FSL’s tools (RobustFOV, BET2) (10). **(C)** ULF FLAIR scans were nonlinearly registered to the down-sampled HF FLAIR using the ANTs multivariate template construction tool (11). The transformation matrix was applied to the non-brain extracted but up-sampled ULF images using nearest neighbor interpolation. **(D)** The ULF images were Fast Fourier transformed and the missing high-frequency components in the under-sampled direction of the ULF axial FLAIR was replaced with that from the ULF coronal FLAIR. The new frequencies were inversely Fast Fourier transformed to image space.

Same-day paired MRI data were acquired using ULF and HF scanners (Figure 2A) on MRI phantoms and participants of the Natural History of MS Study protocol at the NIH. Ethical approval was obtained from the institutional review board at the NIH (NCT00001248), and all participants provided written informed consent.

The ULF data were obtained using the 64mT Hyperfine SWOOP system (software version: rc8.6.0) and included two ULF FLAIR images in the axial (3D FLAIR, TR = 4 s TE = 166.72, TI = 1,426 ms, scan time = 10.15 min with an in-plane resolution of 1.7 mm × 1.7 mm and a slice thickness of 5 mm) and coronal (3D FLAIR, TR = 4,000 ms, TE = 166.72 ms, TI = 1,426 ms, scan time = 8.38 min with an in-plane resolution of 1.7 mm × 1.7 mm and a slice thickness of 5 mm) directions (ULF coronal, ULF axial). The HF data were acquired on a 3 T Siemens scanner and followed a standardized protocol, which included a FLAIR (3D FLAIR, TR = 4,800 ms TE = 352 ms, TI = 1800 ms, scan time = 7 min with 1 mm isotropic resolution) and a T1-weighted (MP2RAGE, TR = 5,000 ms TE = 288, TI_1_ = 500, TI_2_ = 900, scan time = 6 min with 1 mm isotropic resolution) sequence.

### 3.2 Image processing

Following data acquisition, the HF and ULF data were preprocessed (Figure 2B). All data were resampled to 1.7 mm isotropic resolution and zero-padded to uniform dimensions. *In vivo* data were skull-stripped to remove non-brain tissue using FSL’s (FMRIB Software Library) BET tool for the HF images (10) and SynthStrip for the ULF images (9). ULF images were nonlinearly registered to the down sampled HF images, using the Advanced Normalization Tools (ANTS) multivariate template construction tool (12) (Figure 2C). The obtained transformation matrices were then applied to the non-brain extracted images. All image transformations were interpolated using nearest-neighbor interpolation and then normalized to the 98th percentile to preserve original voxel intensities while mitigating the impact of extreme intensity outliers. These preprocessed ULF images acquired in the coronal and axial plane will be referred to as the ULF coronal and ULF axial, and their average will be referred to as the “ULF average.”

MRI SR methods leverage the high-frequency details inherent in multiple low-resolution images to construct a single higher-resolution image. In the FouSR approach, the missing high-frequency components in the under-sampled direction of the ULF coronal were replaced with those from the ULF axial. The fast Fourier transform was applied to the preprocessed ULF coronal and ULF axial scans (Figure 2D), and in frequency space, the inner 64 points of the ULF coronal were replaced with those of the ULF axial. To determine the amount of information that needs to be recovered in the under-sampled direction, the ratio of the through-slice thickness (5 mm) to the in-plane resolution (1.7 mm) was used. This calculation results in a 2.94-fold increase in resolution compared to the original through-plane resolution. The factor of 2.94 was used to determine the number of frequency space lines to be replaced along the axial direction, which was determined to be 54 of 160. However, after conducting stepwise replacements ranging from 50 to 70 lines, it was found that replacing 64 lines yielded the optimal results, likely due to the higher noise components associated with outer regions of the frequency-space. The new frequencies were then inversely fast Fourier transformed to create the image.

FouSR images were also compared to another SR approximation described by Niaz et al. (11), which follows a similar iterative registration and resampling process of multiple acquisitions but also incorporates iterative Laplacian sharpening before image combination (ANTs-SR). To generate ANTs-SR images, *in-vivo* data without skull-stripping were first resampled to 0.7 mm isotropic and then processed using the antsmultivariatetemplateconstruction.sh tool, with the HF FLAIR image serving as the reference volume for registration. Laplacian filtering was applied at each iteration while all remaining settings were maintained at their default values. Finally, images were normalized to their 98th percentile.

### 3.3 Quantitative analysis

Regions of interest (ROI) were manually annotated in the high signal and background (avoiding any regions with artifacts) areas in ULF coronal, ULF axial, ULF average, and FouSR images from the phantom. On *in vivo* data, brain masks were obtained from the down-sampled HF FLAIR (Figure 2B) and multiplied by the ULF coronal, ULF axial, ULF average, FouSR, and ANTs-SR images. Manual WML masks were annotated on the down-sampled HF FLAIR images and used to calculate the total WML volume. The total WML mask was spatially split into individual clusters for individual lesion analysis. White matter masks were created from HF T1-weighted images using FSL’s FAST tool (13) and then linearly registered to the down-sampled HF FLAIR. Total WML masks were subtracted from the white matter masks to create normal-appearing white matter (NAWM) ROI. An ROI was manually drawn for each participant on ITKsnap version 3.8.0 (14) to measure background noise.

### 3.4 Data analysis and statistics

In phantom data SNR and intensity plots across three planes for ULF coronal, ULF axial, ULF average, and FouSR were compared. For quantitative evaluation of *in-vivo* data, the SNR, CNR, and variance of the Laplacian (3) were compared. The SNR calculation was measured as the ratio of the mean total lesion signal to the standard deviation of noise. CNR was calculated as the ratio of the absolute difference between mean total lesion and mean NAWM signals relative to the standard deviation of background noise. Lesion conspicuity was calculated as the ratio of the difference between mean lesion and mean NAWM intensity and their sum (3). SNR, CNR and lesion conspicuity are related image quality measures useful for evaluating the visibility of MS lesions and have been used in a previous ULF study (3). Higher SNR indicates a stronger and clearer signal from lesions while higher CNR and lesion conspicuity indicate enhanced separation of lesions from normal brain tissue. SNR and CNR but not lesion conspicuity are measured in relation to background noise.

Image sharpness was quantified using the variance of the Laplacian, computed by applying a 3D Laplace filter from the scipy library in Python (15) and then extracting the standard deviation as a measure of variance (3). The Laplacian operator is a mathematical filter that enhances signal variations in an image, such as the boundaries of different tissues or structures. Higher Laplacian variance signifies increased image intensity variations, characteristic of sharper borders within images. Conversely, in the presence of blurring or reduced sharpness, image edges exhibit less abrupt intensity changes resulting in a lower Laplacian variance. Image sharpness was calculated for skull-stripped data and lesion boundary sharpness was assessed by dilating individual lesion masks and calculating Laplacian variance for these ROI (lesion image sharpness).

To gauge clinical impact, three neurologists with experience in MS and neuroimaging conducted qualitative evaluations of the images blinded to the image generation method. Each image was rated according to defined scales presented in Table 1 for the visibility of WML and the sulcal-gyral delineation accuracy of four specific anatomical landmarks, namely the central sulcus, parieto-occipital sulcus, calcarine sulcus, and Sylvian fissure. To ensure the validity of anatomical landmarks and lesion identification, the down-sampled 3 T FLAIR images were made accessible during the qualitative assessment process.

**Table 1:** Description of qualitative ratings of WML and sulci and gyri performed by MS neurologists in 10 participants.

Rating	WML: lesion level	Sulci and gyri: anatomical landmarks^a^
1—Poor	WML not visible	Delineation not possible
2—Acceptable	WML visible but hard to identify	Delineation possible in some regions
3—Good	WML visible and easy to identify	Delineation good in general, with hyperintense artifacts in gyri
4—Superior	WML-to-normal appearing WM contrast similar to 3 T	Sulci-Gyri contrast sharp with delineation similar to 3 T

a Central sulci, Sylvian fissures, parieto-occipital sulci, calcarine sulci.

Statistical analyses were conducted using the GraphPad Prism version 9.3.1, GraphPad software, and R version 3.6.3. Before analysis, adherence to the normality assumption was tested using the Shapiro–Wilk test. Repeated-measure ANOVA was used to discern significant alterations in mean measures across images. In cases of non-normally distributed data, *post hoc* multiple comparisons were performed with Dunn’s method, and the q ratio was reported. For normally distributed data, Tukey’s *post hoc* multiple comparison test was employed, and *Z* statistic was reported. An established significance threshold of a corrected *p*-value of 0.05 or lower was used to identify statistically significant outcomes. We used linear mixed-effects models nested for participants to compare image sharpness for individual lesions across different images (LME4 package in R). Pairwise comparisons were computed using R’s Emmeans package. Unless stated otherwise, all reported data are presented as mean ± standard deviation.

## 4 Results

### 4.1 Participant demographics

The participant cohort consisted of 10 adults (mean ± SD 44.1 ± 4.1 years old, 6 women) who had been clinically diagnosed with MS, clinically isolated syndrome (CIS), or suspected MS. (16) Within this cohort, 7 individuals presented with relapsing-remitting MS, 1 with suspected MS, and 2 participants exhibited clinically isolated syndrome (CIS). Participants had varying amounts of total lesion burden (4,313 mm^3^ ± 10,026).

### 4.2 Phantom scan

**Figure 3:**
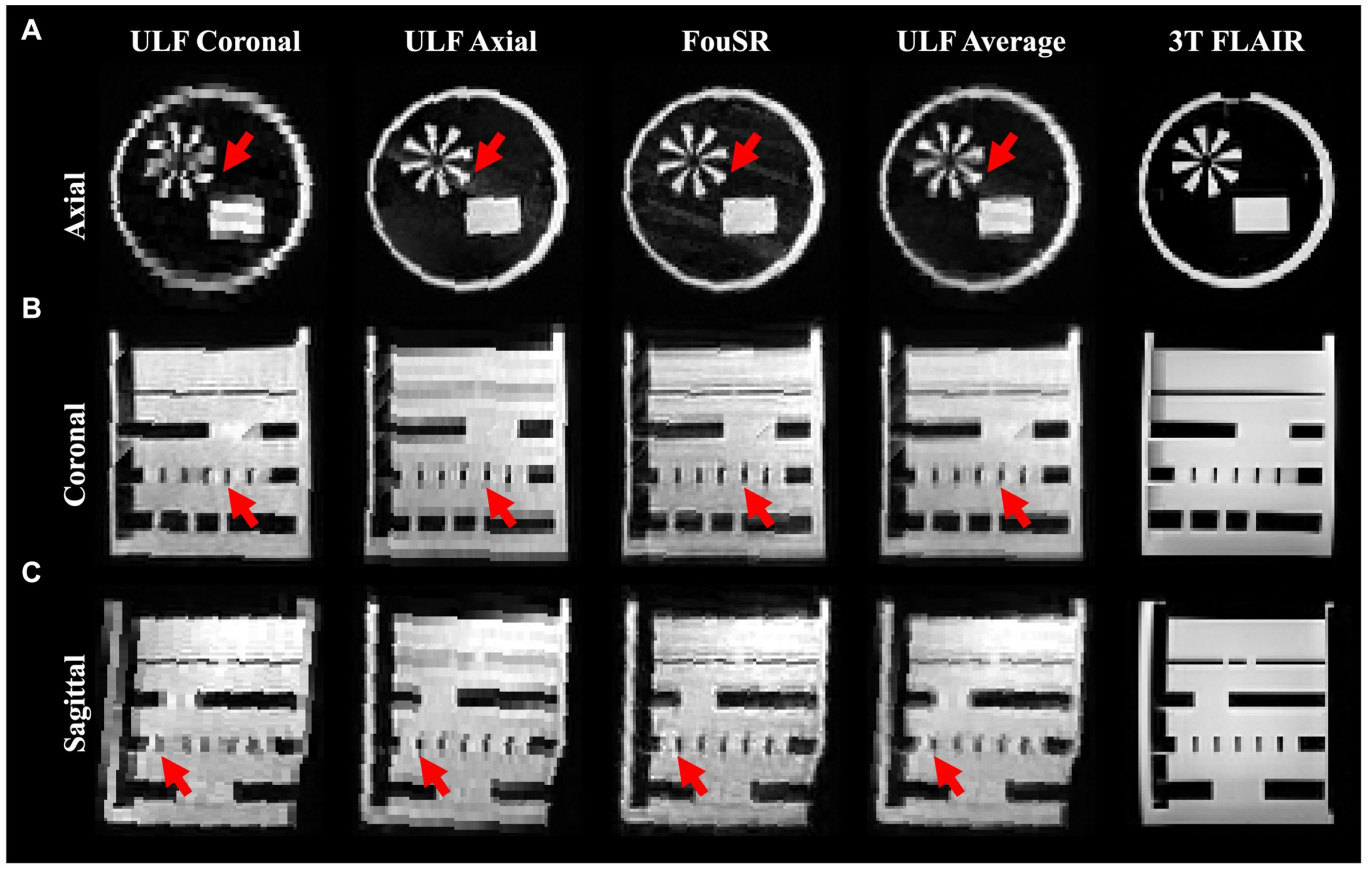
In a standard Hyperfine phantom, FouSR shows improved edge sharpness and image quality compared to ULF FLAIR scans. Representative **(A)** axial, **(B)** coronal, and **(C)** sagittal slices (either directly acquired or reformatted) are shown for ULF coronal FLAIR, ULF axial FLAIR, FouSR, ULF average, and 3 T FLAIR (left to right). Red arrows highlight an example area across each plane where FouSR demonstrates improved resolution and image sharpness compared to other methods.

**Figure 4:**
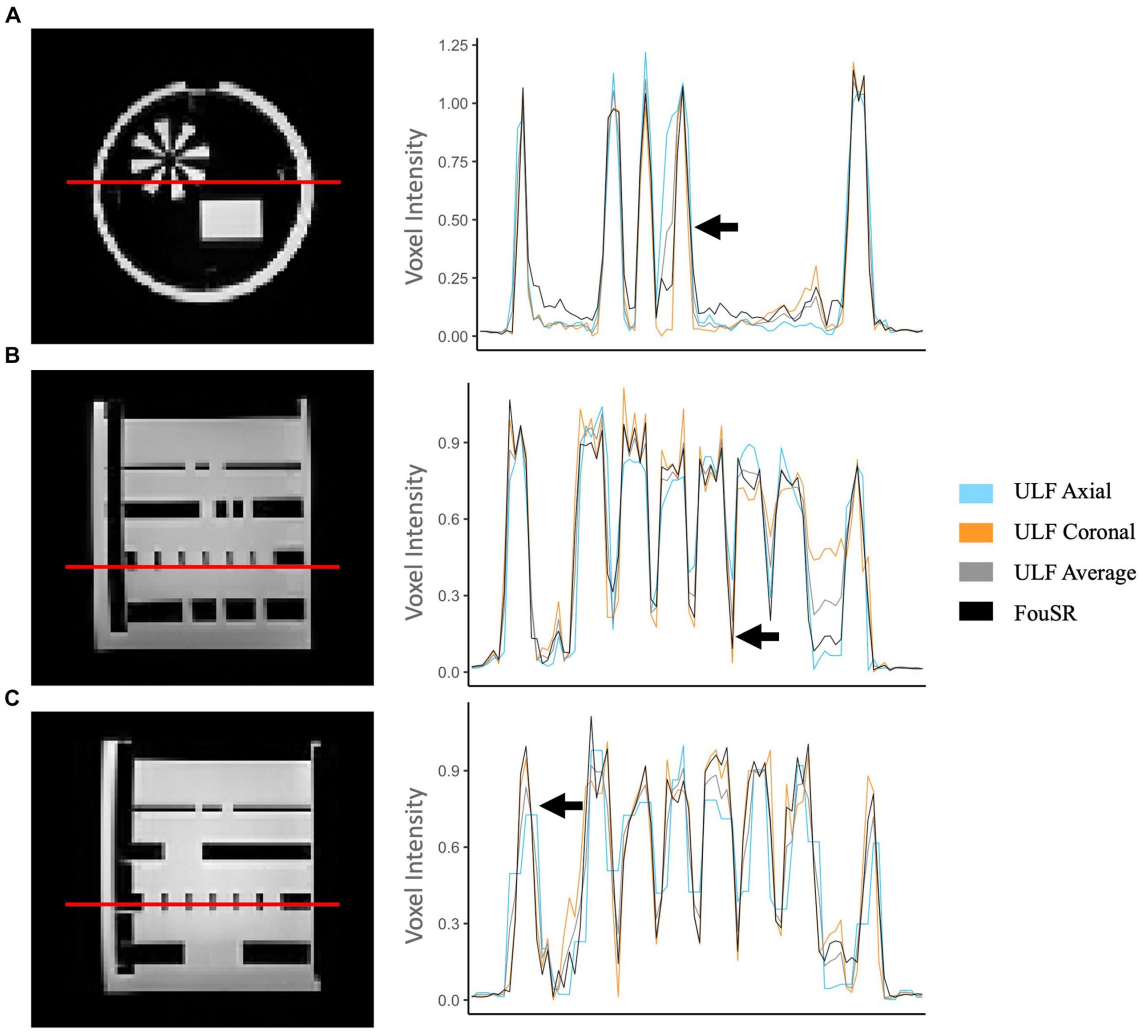
FouSR improves image quality across all three planes. Qualitative visualization of **(A)** axial, **(B)** coronal, and **(C)** sagittal slices in an MS case with high lesion burden in ULF FLAIR images, the average of ULF FLAIR images, after FouSR algorithm and after ANTs SR algorithm is shown. Arrows point to artifacts from partial voluming along the slice direction that is seen in the coronal and axial ULF (orange arrows) but reduced in FouSR and average images (yellow arrows) and absent in ANTs-SR images (green arrows).

Intensity profile plots from ULF coronal, ULF axial, ULF average, and FouSR images of a standard Hyperfine phantom were compared. FouSR recovered information from the ULF axial, leading to sharper shape boundaries (Figure 3A) which are visible as sharper slopes in intensity plots (Figure 4A) while effectively preserving the signal intensity of the ULF coronal (Figures 3B, 4B). Both ULF average and FouSR images exhibited comparable signal intensity in areas of high signal to the in-plane ULF scan (Figure 3B). In the sagittal plane, FouSR images not only better delineated edges in the phantom scan (Figure 3C) but also demonstrated higher signal intensity than in the ULF axial, ULF coronal and ULF average images (Figure 4C). Calculated using manually annotated ROI, FouSR had lower SNR (377) compared to ULF average (614), ULF coronal (389), and ULF axial (494). Overall, the SR algorithm combined the information from both ULF in-plane scans, thereby retaining crucial details without losing sharp delineations that could otherwise be blurred by averaging the two slices.

### 4.3 In-vivo

**Figure 5:**
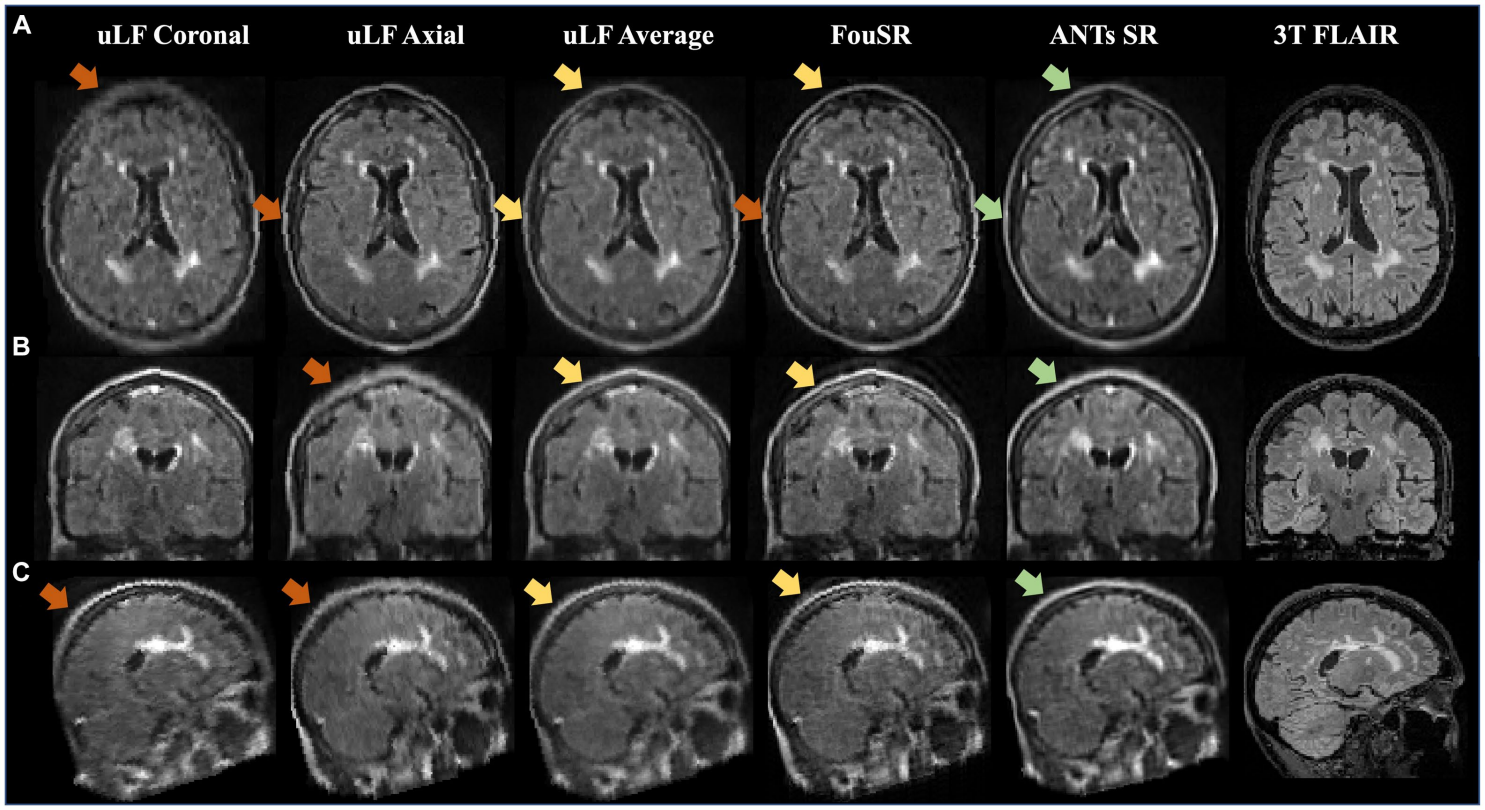
FouSR improves image quality across all three planes. Qualitative visualization of **(A)** axial, **(B)** coronal, and **(C)** sagittal slices in an MS case with high lesion burden in ULF FLAIR images, the average of ULF FLAIR images, after FouSR algorithm and after ANTs SR algorithm is shown. Arrows point to artifacts from partial voluming along the slice direction that is seen in the coronal and axial ULF (orange arrows) but reduced in FouSR and average images (yellow arrows) and absent in ANTs-SR images (green arrows).

Figure 5 presents an example of a participant with a high lesion burden. Notably, FouSR in Figure 5, column 3, shows enhanced resolution across all three axes, surpassing the through-plane resolution of the ULF coronal and ULF axial scans, suggesting the recovery of information from the in-plane voxels of the ULF coronal (Figure 5, column 1) and ULF axial images (Figure 5A, column 2). FouSR demonstrated improved image quality relative to the ULF coronal and ULF axial images in the slice direction. ULF average and ANTs displayed more pronounced blurring along lesion edges and sulci than the FouSR approach. Importantly, certain artifacts visible in the ULF coronal and axial images (Figure 5C, arrows) were consistently retained in the FouSR and ANTs-SR images, although they exhibited reduced visibility in the ULF average. Additionally, ringing artifacts were introduced in FouSR (Figure 5C, box), which were not visible in other scans.

### 4.4 *In-vivo* quantitative

**Figure 6:**
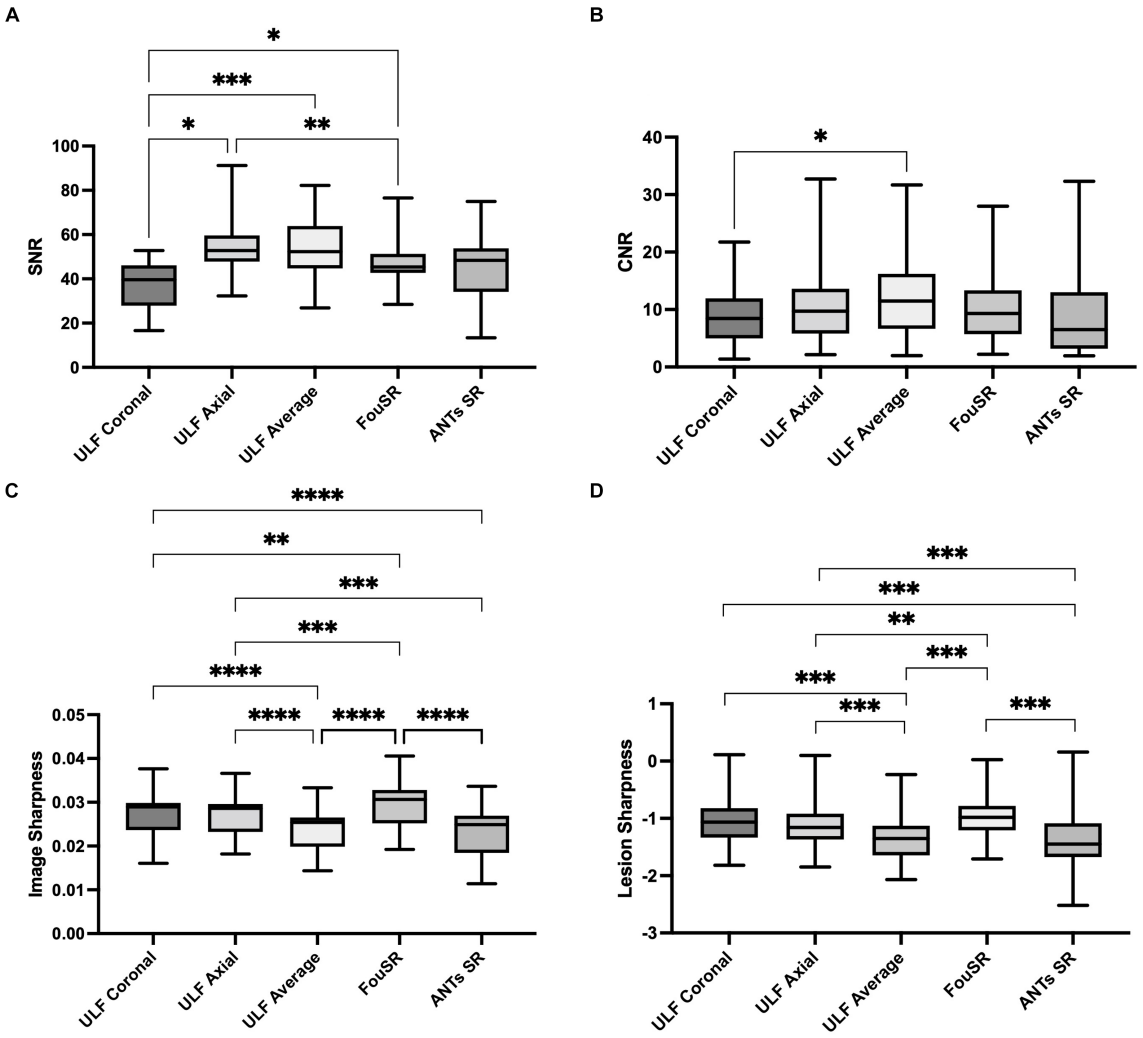
*In-vivo* quantitative assessments of **(A)** SNR, **(B)** CNR, **(C)** image sharpness, and **(D)** lesion sharpness between individual images, average image, FouSR and ANTs-SR in 10 adults with MS show that FouSR produces the sharpest images. (^*^p < 0.05 in pairwise mean difference).

Quantitative comparisons were made between FouSR, ULF coronal, ULF axial, ULF average, and ANTs SR methods by comparing the SNR, CNR, image sharpness, and lesion sharpness. ULF coronal exhibited the lowest SNR at 36.7 ± 12.2, a value significantly lower than ULF axial (54.9 ± 6.0), ULF average (53.2 ± 16.5) and FouSR (48.0 ± 12.6) (Figure 6A; Table 2). ULF axial also had significantly higher SNR compared to FouSR. ULF average had the highest mean CNR (12.5 ± 9.2) but was only significantly higher than ULF coronal (9.2 ± 6.0) (Figure 6B). Lesion conspicuity was higher in FouSR compared to ULF axial (0.11 ± 0.06) but not significantly different than other methods. ULF coronal (0.13 ± 0.07) had significantly higher lesion conspicuity compared to ULF axial, ULF average (−1.4 ± 0.4) but not compared to ANTs SR (−1.52 ± 0.5) (Table 2).

**Table 2:** Mean and standard deviations of quantitative and qualitative measures for each image.

Metric	ULF coronal	ULF axial	FouSR	ULF average	ANTs SR
Qualitative WML ratings	2.3 ±0.76	2.6 ±0.66	2.67 ±0.81	2.5± 0.61	2.68 ± 0.75
Qualitative sulci and gyri ratings	1.67 ± 0.27	2.2 ± 0.40	2.1± 0.32	2.2± 0.33	2.43±0.50
SNR	3 6.7± 12.2	54.9 ± 15.19	47.96 ± 12.56	53.18 ± 16.54	45.3 ± 16.8
CNR	9.2 ± 6.0	11.5± 8.6	10.58 ± 7.43	12.47 ± 8.86	9.6 ± 9.2
Whole brain image sharpness	0.02 8± 0.0056	0.0 27± 0.0052	0.02 9± 0.0058	0.024 ± 0.0052	0.023 ± 0.0062
Lesion sharpness (log)	−1.0 45± 0.35	−1. 14± 0.35	−0.97 ± 0.31	−1.37 ± 0.37	−1.52±0.46
Lesion conspicuity	0.13 ± 0.07	0.11 ± 0.06	0.1 2± 0.056	0.12 ± 0.062	0.11 ± 0.07

FouSR exhibited superior sharpness compared to all other image types (Figure 6C). FouSR displayed an 18 and 23% enhancement in image sharpness compared to ULF average and ANTs SR, respectively. FouSR also displayed a less dramatic but still significantly higher image sharpness than ULF coronal and ULF axial (Figure 6C; Table 2). Lesion sharpness was analyzed using a linear mixed model nested for participants with individual lesions. To adhere to normality assumptions, the lesion sharpness values were log-transformed. FouSR demonstrated notably higher log lesion sharpness (−0.971 ± 0.3) compared to ULF average (−1.37 ± 0.4, *t*(543) = −10.2, *p* = <0.0001). Furthermore, ULF average exhibited lower lesion sharpness in comparison to both ULF axial (−1.14 ± 0.4, *t*(543) = −7.51, *p* < 0.0001) and ULF coronal images (−1.05 ± 0.35, *t*(543) = −8.83, *p* < 0.0001) likely due to boundary-blurring effects from averaging (Figure 6D).

### 4.5 *In-vivo* qualitative

**Figure 7:**
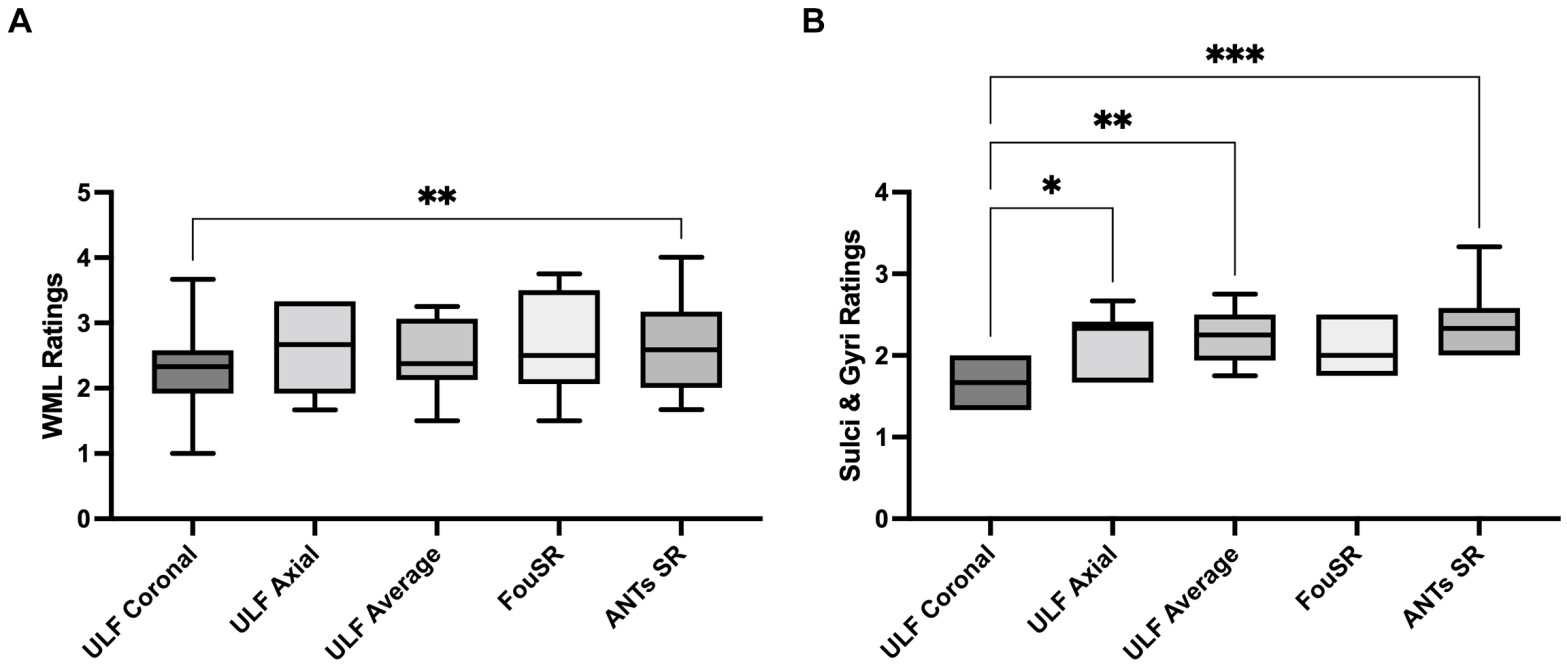
Qualitative ratings for white matter lesions (WML) are highest in ULF average, followed by FouSR and ANTs SR. ANTs SR has higher qualitative ratings of sulci and gyri than ULF coronal. For each method, per participant qualitative rating of **(A)** WML **(B)** sulci and gyri. (^*^p < 0.05, pairwise mean difference).

ANTs SR had the highest mean WML ratings (2.68 ± 0.78) followed by FouSR (2.67 ± 0.81) and ULF axial (2.2 ± 0.40) (Figure 7A). Only ANTs SR showed a significant difference from ULF coronal (2.3 ± 0.76). ANTs SR (2.43 ± 0.50), ULF average (2.2 ± 0.33) and ULF axial (2.2 ± 0.40) had significantly higher sulci and gyri ratings compared to ULF coronal (1.65 ± 0.27) (Figure 7B). FouSR (2.1 ± 0.32) had a comparable mean sulci and gyri rating and was not significantly different from other methods. While FouSR exhibited superior sharpness, ULF average and ANTs SR maintained more uniform intensities within tissue categories, particularly in the WM. This uniformity in intensity is crucial for the visual identification of anatomical landmarks and lesions. It is worth noting that while ANTs-SR may not be easily implementable on the scanner, it does offer a visually appealing smoothing effect.

## 5 Discussion

In this study, we present FouSR, an SR algorithm designed to enhance the resolution of an ULF MRI scan by leveraging high-frequency information in an additional orthogonal scan direction. The images reconstructed using FouSR had superior boundary sharpness compared to single-plane acquisitions and an average of two full scans in orthogonal directions while maintaining comparable quantitative image metrics such as SNR and CNR in patient scans. While the SR algorithm is described here as a post-acquisition image process, it can be implemented on the scanner during acquisition. If implementing this technique on the scanner, the additional orthogonal scan only needs to sample the high-frequency components, potentially yielding ~3× improvement in slice resolution with a proposed ~60% increase in scan time relative to single plane-acquisition (20% less scan time than 2 full orthogonal scans). Additionally, the overall data size would only increase 40% compared to a current single acquisition and decrease by 33% compared to two full acquisitions. Such high-resolution techniques can lead to better visualization of small focal abnormalities, leading to diagnosis without the need for a high-field scan.

Previous studies have explored deep learning (DL) approaches to enhance ULF image resolution and quality (17–20). Learning-based SR leverages convolutional neural networks (CNNs) to capture intricate patterns and relationships within data, thereby generating high-resolution images from low-resolution inputs. Learning-based SR can offer more than just improved spatial resolution for ULF images; DL models can also learn to mitigate noise and Gibbs ringing artifacts (19) and mimic HF contrast (18). However, these approaches encounter several significant challenges. Notably, the scarcity of large, paired datasets comprising HF and ULF images compels studies to rely on HF data for training (17–20). Despite attempts to simulate ULF conditions through down-sampling and noise addition, such models may fail to capture the unique noise, contrast, and spatial distortions inherent to ULF MRI. Furthermore, while some deep learning SR methods have shown success in aging or specific diseases, adapting these models to new disease types necessitates substantial, disease-specific dataset acquisition, limiting their generalizability and clinical implementation. In contrast, FouSR does not rely on deep learning or synthetic data generation. Instead, it derives all information directly from existing ULF acquisitions, thus enhancing its potential for generalizability across various ULF MRI contrasts and diseases. Modifications in the SR algorithm should be limited to the frequency space sampling scheme and may be needed only when acquisition resolution is modified.

Accurate coregistration of the orthogonal ULF images is paramount for the success of FouSR, and failures can impede the visualization of anatomical details. Specific decisions were made to optimize the registration process, including registration to HF references, the choice of registration tools, and skull-stripping before registration. Registering to paired, same-day HF provided an unbiased reference space, enabling direct comparison of anatomical structures without additional processing. It’s essential to note that the 3 T scan is not a mandatory component for the algorithm; the ULF data could have been registered to each other or to a halfway space, and the algorithm would still function. Indeed, our goal was to improve the resolution of the ULF scans and compare FouSR to a clinical routine scan at 3 T, which serves as a standard sequence for detecting white matter pathology. This choice allows us to evaluate our algorithm within the context of what is conventionally available and widely employed in clinical practice, offering valuable insights into its performance relative to established standards.

The ANTs multivariate template construction method was used to achieve optimal registration herein (12). It was selected for its superior performance in aligning ULF images to HF references in our experience and its previous use in ULF SR studies (5). Skull-stripping was achieved using SynthStrip on ULF scans (9). However, this approach has prolonged processing times and substantial computational demands, with non-parallel execution potentially exceeding 40 min, which may not align with clinical implementation requirements. Alternative faster registration and skull-stripping techniques were also explored, but they failed to match the quality achieved with ANTs and SynthStrip for data reported. These challenges align with previous reports on the inherent difficulties of processing ULF MRI scans, partly due to the predominant optimization of open-source MRI software tools for more conventional 1.5 T or 3 T MRI systems (5, 18).

FouSR, the ULF average, and ANTs SR all use the ANTs multivariate template construction tool to varying degrees (12). Specifically, FouSR and the ULF average employ this tool solely for obtaining an optimized registration matrix. ANTs SR is the tool’s default output, which involves the inversion of the average diffeomorphism and the blurring induced by intensity averaging, a technique referred to as “soft sharpening.” As visually demonstrated, ANTs SR yields more uniform intensity values within tissue types, a characteristic reminiscent of the ULF average, and bears a closer resemblance to HF scans, where the clear demarcation of homogenous intensity values between white matter and lesions provides a distinct advantage in lesion detection. ANTs SR’s incorporation of a sharpening filter enhances the delineation of lesion edges, a feature lost in the ULF average. ANTs SR’s interpolation and blurring effects play a pivotal role in mitigating artifacts present in the original ULF scans. This contrasts with FouSR, where artifacts and the utilization of nearest neighbor interpolation can bolster signal-to-noise ratio and sharpness but may inadvertently introduce intensity heterogeneity in WM regions, posing a challenge in distinguishing noise from subtle anatomical features such as small lesions.

FouSR has limitations. FouSR displayed higher occurrence of artifacts compared to other images: predominantly within-tissue granularity and Gibbs ringing (Figure 5). Importantly, at the level of acquisition the direction of the phase and slice encoding directions were different in the ULF coronal and ULF axial, resulting in discrepancies in off-resonance components, distortions and B0 field inhomogeneities between the two images. Given the naïve combination of ULF coronal and axial images, FouSR is highly sensitive to spatial and intensity inconsistencies between the two images, with any mismatches potentially resulting in undesired artifacts.

Given the algorithm’s reliance on registration quality, distortion and motion artifacts may compromise the overall output. *In-vivo* ULF acquisitions displayed noticeably more distortions, mainly in the occipital area, compared to high-field scans. Importantly, the distortions seen in the coronal and axial acquisitions were dissimilar, largely due to the swapping of the phase and slice encode directions. ULF phantom acquisitions portrayed minimal distortion artifacts, mostly attributed to their smaller size compared to a typical human head, and placement at the center of the DSV away from field inhomogeneities. However, distortions in *in-vivo* scans sometimes degraded the quality of nonlinear registrations. Any residual mismatches between the orthogonal scans used in FouSR caused ringing artifacts in SR outputs. Furthermore, these artifacts were less conspicuous in the ULF average, likely due to the smoothing effect of averaging at the expense of boundary sharpness.

Additionally, the process of image combination in FouSR was performed in the frequency space after zero-padding, interpolation to isotropic resolution and registration to a paired HF scan. However, spatially transforming an image matrix before Fourier transformation can diminish image quality as interpolation modifies the spatial distribution of voxels, altering the frequency content. Methods like zero-padding and nearest-neighbor interpolation may introduce abrupt transitions between voxels, leading to unwanted artifacts within the Fourier domain. This manifested in the FouSR outputs as pronounced Gibbs ringing and noticeable granularity.

B0-maps and navigator-based motion correction could improve the SR outputs, especially in the proposed on-scanner reconstruction (21). While B0-maps can be acquired to correct distortions post-acquisition, acquiring and reconstructing FouSR images on the scanner during data acquisition by manipulating the *k*-space trajectory, and using navigator scans for on-the-fly motion corrections would better address many of FouSR’s current limitations. Implementation of the FouSR algorithm on the Hyperfine System could be achieved through modification of the *k*-space trajectory. Due to the proprietary nature of the scanner interface used, this currently must be achieved through collaboration with the manufacturer. Online implementation will be explored in future work.

Integrating FouSR during data acquisition would not only address the post-processing interpolation-related limitations, but also enable the integration of navigator-based corrections of *k*-space data before image reconstruction and SR combination. Navigators, short sequence elements incorporated into the image sequence within the echo time, offer real-time measurements of motion and distortion during scanning (21–25). This real-time data processing allows for immediate adjustments to the imaging system and sequence, reducing the occurrence of artifacts caused by dynamic perturbations, thereby enhancing overall image quality (21, 26). Future research aims to investigate the application of navigators in the ULF SR.

FouSR uses a 3D-Fourier transformation to combine two images acquired orthogonally. In the case of the ULF scanner, standard 3D sequences are acquired with anisotropic voxels to provide the optimal image quality for standard radiological reading with limited available SNR. This provides an opportunity to acquire an orthogonal image, the high in-plane resolution of which would compensate for the low resolution along the slice direction of the other. While alternative sampling schemes after registration were not investigated here, the amount sampled seemed within an optimal range for many quantitative measures (data not shown). However, there is a need for further exploration with a larger participant cohort.

Keyhole-imaging technique is another sampling method that, similarly to FouSR, combines frequency data from two acquisitions. The Keyhole technique was originally proposed to accelerate the acquisition of dynamic contrast enhanced (DCE) images while maintaining a reasonable level of resolution (8). In this approach, the central region of *k*-space, termed the “keyhole, “is sampled more frequently and combined with the outer regions of a fully sampled reference image, facilitating say, rapid imaging of bolus passage, leading to better characterization of perfusion parameters. Indeed, such methods have been combined with accelerated imaging methods such as propeller for even faster time-resolution (8, 27–33). The key difference between keyhole and FouSR is that the second scan of FouSR is in an orthogonal plane to capture high-frequency components missing in the original image to improve its spatial resolution. Other examples of frequency domain combinations would include acceleration techniques such as GRAPPA, in this case from various elements of an array coil.

This study has limitations. The small sample size, specifically chosen for assessing small WML visualization, hinders the generalizability of the findings. Using downsampled images and potential registration inaccuracies may compromise the precision of key metrics like SNR, CNR, and lesion conspicuity, especially for smaller lesions. In addition, the equations for calculating CNR and SNR necessitate the standard deviation of the noise, which was derived from an ROI drawn on the background of scans, as is the standard practice. Variability in the noise measure can arise, especially in the *in-vivo* scans, from artifacts in the phase and slice directions, which are different in different images. Furthermore, the compress-SENSE acquisition scheme on the scanner gives rise to regions in the image background that are devoid of noise. ROIs were carefully placed to avoid such regions. Additionally, potential morphological changes introduced by nonlinear registration procedures pose challenges to the generalizability of the study’s outcomes.

In conclusion, our study presents preliminary findings that show the potential of our FouSR algorithm in enhancing the resolution of ULF scans. Although our results are based on a phantom scan and a limited cohort of MS cases, FouSR showed enhanced delineation of MS lesions and other significant anatomical features compared to single scans. Such enhancements in lesion delineation could improve the ability of ULF MRI to detect and monitor the subtle lesional changes characteristic of MS, crucial for both diagnosis and longitudinal monitoring. We highlight the feasibility and benefits of implementing the SR algorithm directly on the MRI scanner. Overall, our SR algorithm holds substantial promise in augmenting the clinical utility of ULF scans.

## Data availability statement

The raw data supporting the conclusions of this article will be made available by the authors, without undue reservation and in compliance with NIHs data sharing policies.

## Ethics statement

The studies involving humans were approved by Institutional Review Board at the NIH (NCT00001248). The studies were conducted in accordance with the local legislation and institutional requirements. The participants provided their written informed consent to participate in this study.

## Author contributions

CD: Data curation, Formal analysis, Methodology, Writing – original draft, Writing – review & editing. SO: Writing – review & editing. CT: Formal analysis, Validation, Writing – review & editing. MG: Formal analysis, Validation, Writing – review & editing. MP: Writing – review & editing. DR: Writing – review & editing. GN: Writing – original draft, Writing – review & editing.
